# Microbial biocontrol agents. An annotated selection of World Wide Web sites relevant to the topics in Microbial Biotechnology

**DOI:** 10.1111/1751-7915.12065

**Published:** 2013-06-11

**Authors:** Lawrence P Wackett

**Affiliations:** & Department of Biochemistry, Molecular Biology & Biophysics, BioTechnology Institute, University of MinnesotaSt. Paul, MN, 55108, USA

## The microbial world: Biocontrol

http://archive.bio.ed.ac.uk/jdeacon/microbes/control.htm

This interesting webpage goes into details about several microbial control agents. These include *Bacillus thuringiensis*, a non-infectious *Agrobacterium tumefacians* as a control for crown gall disease and several fungal biocontrol agents.

## Biocontrol; Microbe wiki

http://microbewiki.kenyon.edu/index.php/Biocontrol

This site provides a broad definition of microbial biocontrol agents and gives several examples of these agents.

## Bacillus thuringiensis

http://www.bt.ucsd.edu/index.html

This site focuses on the use of *B. thuringiensis* in agriculture and has nice graphics, some animations and links to further information.

## Association of natural biocontrol producers

http://www.anbp.org

This website is put up by the professional trade organization of manufacturers of biocontrol pest management agents. It contains information pertaining to news, events and new research findings.

## International biocontrol manufacturers association

http://www.ibma.ch/pg_microorganisms_mbca.html

This website is an international association dedicated to the proper use of biocontrol agents for the benefit of industry and society.

## Beneficial fungal parasites

http://www.angelfire.com/wizard/kimbrough/Textbook/FungiAsBiocontrolAgents_blue.htm

This site discusses many crop diseases and how they can be controlled biologically. It contains many useful photographs to illustrate the disease-causing agents, the plants they attack, and the fungi that control the diseases.

## Biological control: A guide to natural enemies

http://www.biocontrol.entomology.cornell.edu

This site contains information, photographs and links relevant to biological control agents commonly used in North America.

## Biological control agents used in New Zealand

http://www.epa.govt.nz/new-organisms/popular-no-topics/Pages/biological-control-agents.aspx

This site contains information on insect and fungal biocontrol agents approved for usage in New Zealand.

## EPA's regulation of *Bacillus thuringiensis* (Bt) crops

http://www.epa.gov/pesticides/biopesticides/pips/regofbtcrops.htm

This webpage details the history of EPA regulatory actions on Bt crops used in the United States.

## National center for integrated pest management

http://www.cipm.info

This site is dedicated to fostering integrated pest management practices that includes the use of microbial biocontrol agents.

## Locust control with fungi

http://en.wikipedia.org/wiki/LUBILOSA

This site describes a research program focused on developing a biological control, typically via fungi, for locust infestations.

## Microbial insecticides

http://bioenergy.asu.edu/photosyn/courses/bio_343/lecture/insect.html

This chapter from a course contains information on microbial biocontrols for insects.

## Biopesticide: Wikipedia

http://en.wikipedia.org/wiki/Biopesticide

This site defines biopesticides as living agents, typically bacteria or fungi, or natural product chemicals that control pests.

## Biological pest control

http://en.wikipedia.org/wiki/Biological_pest_control

While this Wikipedia page appears similar in name to the one above, it offers a different perspective, dealing with biological pest control from an ecological standpoint.

